# (3*S*,4*R*)-4-(4-Fluoro­phen­yl)-3-(hydroxy­meth­yl)piperidinium chloride^1^

**DOI:** 10.1107/S1600536808008593

**Published:** 2008-04-04

**Authors:** M Nirmala, B. R. Sreekanth, Peddy Vishweshwar, J. Moses Babu, Y Anjaneyulu

**Affiliations:** a Department of Analytical Research, Discovery Research, Dr Reddy’s Laboratories Ltd, Miyapur, Hyderabad 500 049, India; b Centre for Atmospheric Science, Jawaharlal Nehru Technological University, Hyderabad 500 072, India

## Abstract

The title compound, C_12_H_17_FNO^+^·Cl^−^, is a degradation impurity of paroxetine hydro­chloride hemihydrate (PAXIL), an anti­depressant belonging to the group of drugs called selective serotonin reuptake inhibitors (SSRIs). Similar to the paroxetine hydro­chloride salt with protonation having taken place on the basic piperidine ring, the degradation impurity also exists as the hydro­chloride salt. The cyclic six-membered piperidinium ring adopts a chair conformation with the hydroxy­methyl and 4-fluoro­phenyl groups in the equatorial positions. The ions form a tape along the *b* axis through charge-assisted N^+^—H⋯Cl^−^ hydrogen bonds; these tapes are connected by O—H⋯Cl^−^ hydrogen bonds along the *a* axis.

## Related literature

For related literature, see: Bower *et al.* (2007 ▶); de Gonzalo *et al.* (2001 ▶); Barnes *et al.* (1988 ▶); Ibers (1999 ▶).
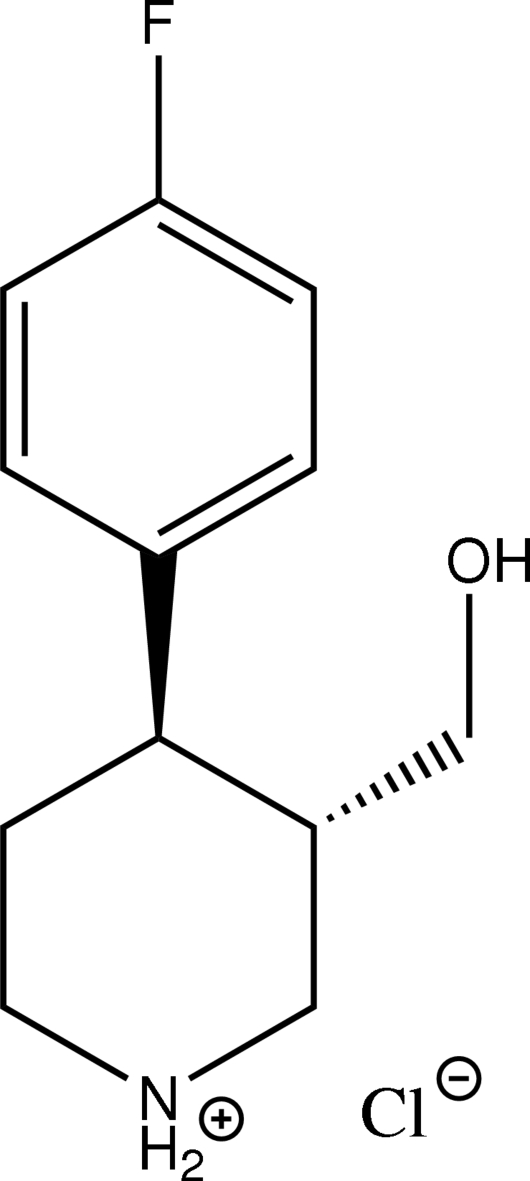


## Experimental

### 

#### Crystal data

C_12_H_17_FNO^+^·Cl^−^
*M* *_r_* = 245.72
Monoclinic, 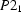

*a* = 7.697 (4) Å
*b* = 5.958 (3) Å
*c* = 13.393 (8) Å
β = 95.505 (5)°
*V* = 611.4 (6) Å^3^
*Z* = 2
Mo *K*α radiation
μ = 0.30 mm^−1^
*T* = 298 K
0.50 × 0.40 × 0.20 mm

#### Data collection

Rigaku Mercury diffractometer
Absorption correction: multi-scan (Jacobson, 1998 ▶) *T*_min_ = 0.863, *T*_max_ = 0.939
6813 measured reflections
2421 independent reflections
2163 reflections with *F* ^2^ > 2σ(*F* ^2^)
*R*_int_ = 0.036

#### Refinement

*R*[*F* ^2^ > 2σ(*F* ^2^)] = 0.065
*wR*(*F* ^2^) = 0.203
*S* = 1.13
2421 reflections
158 parameters
H atoms treated by a mixture of independent and constrained refinement
Δρ_max_ = 0.54 e Å^−3^
Δρ_min_ = −0.37 e Å^−3^
Absolute structure: Flack (1983 ▶), 938 Friedel Pairs
Flack parameter: −0.11 (13)

### 

Data collection: *CrystalClear* (Pflugrath, 1999 ▶); cell refinement: *CrystalClear*; data reduction: *CrystalStructure* (Rigaku/MSC, 2006 ▶); program(s) used to solve structure: *SHELXS97* (Sheldrick, 2008 ▶); program(s) used to refine structure: *SHELXL97* (Sheldrick, 2008 ▶); molecular graphics: *X-SEED* (Barbour, 2001 ▶); software used to prepare material for publication: *CrystalStructure*.

## Supplementary Material

Crystal structure: contains datablocks global, I. DOI: 10.1107/S1600536808008593/tk2259sup1.cif

Structure factors: contains datablocks I. DOI: 10.1107/S1600536808008593/tk2259Isup2.hkl

Additional supplementary materials:  crystallographic information; 3D view; checkCIF report

## Figures and Tables

**Table 1:** Hydrogen-bond geometry (Å, °)

*D*—H⋯*A*	*D*—H	H⋯*A*	*D*⋯*A*	*D*—H⋯*A*
O1—H1⋯Cl1^i^	0.81 (7)	2.35 (7)	3.114 (4)	160 (6)
N1—H11⋯Cl1	0.83 (5)	2.56 (5)	3.234 (5)	140 (4)
N1—H12⋯Cl1^ii^	0.84 (5)	2.41 (5)	3.144 (5)	147 (6)

Symmetry codes: (i) 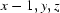; (ii) 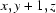.

**Fig. 1.**
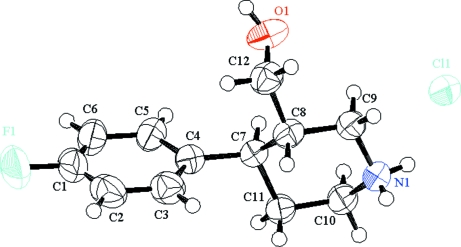
Molecular structure of (I) showing the atom numbering scheme. The displacement ellipsoids are drawn at the 50% probability level. H-atoms are shown by small circles of arbitrary radii.

**Fig. 2.**
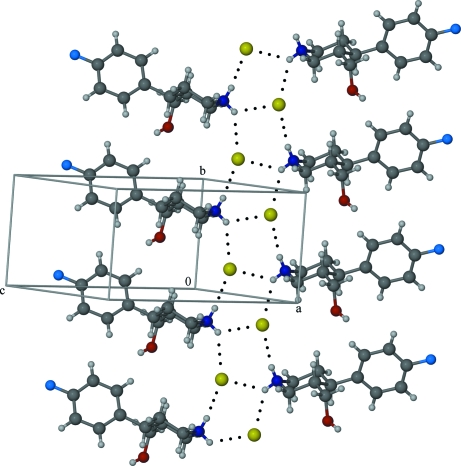
Crystal packing for (I). The molecular tape is sustained through the charge-assisted N+—H···Cl hydrogen bonds, shown as dashed lines.

## supplementary crystallographic information

### Comment

The title compound (I), is a degradation impurity of paroxetine hydrochloride hemihydrate, an orally administered psychotropic drug (PAXIL) (Barnes *et al.*, 1988). The crystal structure of paroxetine hydrochloride hemihydrate has been reported (Ibers, 1999). Herein, we report the synthesis and crystal structure of (I).

Compound (I) was isolated during degradation studies of paroxetine hydrochloride hemihydrate. The paroxetine drug is available in the market as hemihydrate. However, compound (I) is in the anhydrous form, Fig. 1. Similar to the paroxetine hydrochloride salt with protonation having taken place on the basic piperidine ring, the degradation impurity also exists as a hydrochloride salt. The absolute configurations of C7 and C8 atoms were established as *R* and S, respectively, consistent with paroxetine hydrochloride hemihydrate. The six-membered piperidinium ring is in the usual chair conformation with the hydroxylmethyl and 4-fluorophenyl in equatorial positions. The crystal packing shows the formation of a molecular tape along the *b* axis through the charge-assisted N^+^—H···Cl hydrogen bonds (Fig. 2 and Table 1). The tapes thus formed are connected by O—H···Cl^-^ hydrogen bonds along the *a* axis.

### Experimental

Paroxetine hydrochloride hemihydrate (1.5 gr, 3.5 mmol) was taken in a conical flask and dissolved in acetonitrile and tetrahydrofuran solvent mixture (1:1, 20 ml *v*/*v*). About 80 ml of 3% hydrogen peroxide was added to the solution and stirred at 60 °C for 48 h. Chloroform and water was added to the solution and the organic and aqueous layers were separated using separating flask. Benzene was added to the aqueous layer and the product (I) was isolated by drying the solution. Single crystals were obtained during purification of (I) from chloroform and methanol. The product was characterized by mass spectroscopy (*M*+1 at m/*z* 210) and NMR.

### Refinement

The H atoms bonded to the N and O atoms were located in a difference map and refined isotropically, see Table 1 for distances. The remaining H atoms were positioned geometrically and refined in the riding model approximation with C—H = 0.93 - 0.97 Å, and with *U*(H) set to 1.2*U*_eq_(C).

### Crystal data

**Table:**

C_12_H_17_FNO^+^·Cl^–^	*F*_000_ = 260.00
*M**_r_* = 245.72	*D*_x_ = 1.335 Mg m^−^^3^
Monoclinic, *P*2_1_	Mo *K*α radiation λ = 0.71070 Å
Hall symbol: P 2yb	Cell parameters from 3399 reflections
*a* = 7.697 (4) Å	θ = 1.5–27.4º
*b* = 5.958 (3) Å	µ = 0.30 mm^−^^1^
*c* = 13.393 (8) Å	*T* = 298 K
β = 95.505 (5)º	Block, colorless
*V* = 611.4 (6) Å^3^	0.50 × 0.40 × 0.20 mm
*Z* = 2	

### Data collection

**Table:**

Rigaku Mercury diffractometer	2163 reflections with *F*^2^ > 2σ(*F*^2^)
Detector resolution: 7.31 pixels mm^-1^	*R*_int_ = 0.036
ω scans	θ_max_ = 27.4º
Absorption correction: multi-scan(Jacobson, 1998)	*h* = −9→9
*T*_min_ = 0.863, *T*_max_ = 0.939	*k* = −5→7
6813 measured reflections	*l* = −17→17
2421 independent reflections	

### Refinement

**Table:**

Refinement on *F*^2^	*w* = 1/[σ^2^(*F*_o_^2^) + (0.1198*P*)^2^ + 0.1259*P*] where *P* = (*F*_o_^2^ + 2*F*_c_^2^)/3
*R*[*F*^2^ > 2σ(*F*^2^)] = 0.065	(Δ/σ)_max_ = 0.006
*wR*(*F*^2^) = 0.203	Δρ_max_ = 0.54 e Å^−^^3^
*S* = 1.13	Δρ_min_ = −0.37 e Å^−^^3^
2421 reflections	Extinction correction: none
158 parameters	Absolute structure: Flack (1983), 938 Friedel Pairs
H atoms treated by a mixture of independent and constrained refinement	Flack parameter: −0.11 (13)

### Special details

**Table:**

Geometry. Bond distances, angles *etc*. have been calculated using the rounded fractional coordinates. All su's are estimated from the variances of the (full) variance-covariance matrix. The cell e.s.d.'s are taken into account in the estimation of distances, angles and torsion angles
Refinement. Refinement was performed using all reflections. The weighted *R*-factor (*wR*) and goodness of fit (*S*) are based on *F*^2^. *R*-factor (gt) are based on *F*. The threshold expression of *F*^2^ > 2.0 σ(*F*^2^) is used only for calculating *R*-factor (gt).

### Fractional atomic coordinates and isotropic or equivalent isotropic displacement parameters (Å^2^)

**Table:**

	*x*	*y*	*z*	*U*_iso_*/*U*_eq_	
F1	−0.4169 (4)	1.0551 (8)	0.5003 (3)	0.1111 (13)	
O1	−0.1239 (4)	0.4486 (7)	0.1385 (3)	0.0778 (11)	
N1	0.3679 (4)	0.7472 (8)	0.1251 (2)	0.0576 (10)	
C1	−0.2900 (5)	0.9849 (10)	0.4441 (4)	0.0738 (16)	
C2	−0.2390 (6)	1.1230 (9)	0.3728 (4)	0.0764 (14)	
C3	−0.1118 (5)	1.0490 (8)	0.3135 (3)	0.0651 (12)	
C4	−0.0374 (4)	0.8397 (6)	0.3271 (2)	0.0505 (10)	
C5	−0.0914 (5)	0.7080 (9)	0.4028 (2)	0.0584 (11)	
C6	−0.2175 (5)	0.7774 (10)	0.4632 (3)	0.0715 (16)	
C7	0.0988 (3)	0.7497 (7)	0.2628 (2)	0.0460 (9)	
C8	0.0506 (4)	0.7783 (6)	0.1502 (2)	0.0504 (10)	
C9	0.1889 (4)	0.6685 (7)	0.0934 (2)	0.0545 (11)	
C10	0.4156 (4)	0.7361 (10)	0.2348 (2)	0.0583 (10)	
C11	0.2796 (4)	0.8529 (7)	0.2909 (2)	0.0545 (10)	
C12	−0.1260 (5)	0.6790 (8)	0.1137 (3)	0.0607 (14)	
Cl1	0.50505 (12)	0.23916 (19)	0.10014 (7)	0.0599 (3)	
H1	−0.216 (9)	0.388 (14)	0.144 (5)	0.11 (2)*	
H2	−0.28790	1.26510	0.36350	0.0910*	
H3	−0.07640	1.14260	0.26370	0.0780*	
H5	−0.04120	0.56700	0.41370	0.0700*	
H6	−0.25160	0.68700	0.51450	0.0860*	
H7	0.10910	0.58820	0.27590	0.0550*	
H8	0.04830	0.93910	0.13470	0.0600*	
H11	0.432 (6)	0.658 (9)	0.099 (3)	0.062 (14)*	
H12	0.378 (10)	0.869 (7)	0.095 (5)	0.12 (2)*	
H91	0.18390	0.50730	0.10290	0.0650*	
H92	0.16270	0.69860	0.02230	0.0650*	
H101	0.52820	0.80710	0.25100	0.0700*	
H102	0.42530	0.58030	0.25570	0.0700*	
H111	0.27690	1.01140	0.27430	0.0650*	
H112	0.31030	0.83830	0.36250	0.0650*	
H121	−0.21760	0.75500	0.14550	0.0730*	
H122	−0.14830	0.69780	0.04160	0.0730*	

### Atomic displacement parameters (Å^2^)

**Table:**

	*U*^11^	*U*^22^	*U*^33^	*U*^12^	*U*^13^	*U*^23^
F1	0.0734 (18)	0.145 (3)	0.120 (2)	0.001 (2)	0.0353 (17)	−0.061 (2)
O1	0.0611 (19)	0.076 (2)	0.094 (2)	−0.0245 (16)	−0.0043 (17)	0.0047 (18)
N1	0.0510 (15)	0.0604 (19)	0.0638 (17)	−0.0101 (18)	0.0179 (13)	−0.010 (2)
C1	0.050 (2)	0.095 (4)	0.078 (2)	−0.005 (2)	0.0143 (19)	−0.031 (2)
C2	0.065 (2)	0.066 (2)	0.097 (3)	0.009 (2)	0.002 (2)	−0.026 (2)
C3	0.064 (2)	0.057 (2)	0.074 (2)	0.0021 (19)	0.0046 (19)	−0.002 (2)
C4	0.0491 (17)	0.0488 (19)	0.0537 (17)	−0.0014 (15)	0.0063 (14)	−0.0020 (15)
C5	0.0510 (17)	0.069 (2)	0.0563 (18)	−0.0044 (18)	0.0103 (14)	0.0036 (19)
C6	0.058 (2)	0.097 (4)	0.062 (2)	−0.010 (2)	0.0187 (16)	−0.010 (2)
C7	0.0420 (14)	0.0472 (16)	0.0491 (15)	−0.0047 (16)	0.0056 (11)	−0.0011 (16)
C8	0.0494 (16)	0.052 (2)	0.0494 (16)	−0.0049 (14)	0.0024 (12)	0.0050 (14)
C9	0.055 (2)	0.060 (2)	0.0492 (17)	−0.0112 (15)	0.0088 (14)	−0.0045 (15)
C10	0.0421 (15)	0.070 (2)	0.0624 (19)	−0.004 (2)	0.0035 (13)	−0.011 (2)
C11	0.0438 (17)	0.067 (2)	0.0523 (17)	−0.0049 (16)	0.0025 (13)	−0.0104 (18)
C12	0.052 (2)	0.072 (3)	0.057 (2)	−0.0043 (17)	−0.0006 (15)	0.0019 (18)
Cl1	0.0573 (4)	0.0551 (5)	0.0682 (5)	−0.0063 (4)	0.0106 (3)	−0.0025 (4)

### Geometric parameters (Å, °)

**Table:**

F1—C1	1.355 (6)	C8—C12	1.520 (5)
O1—C12	1.412 (6)	C10—C11	1.515 (5)
O1—H1	0.81 (7)	C2—H2	0.9300
N1—C10	1.481 (4)	C3—H3	0.9300
N1—C9	1.479 (5)	C5—H5	0.9300
N1—H12	0.84 (5)	C6—H6	0.9300
N1—H11	0.83 (5)	C7—H7	0.9800
C1—C6	1.370 (8)	C8—H8	0.9800
C1—C2	1.347 (8)	C9—H91	0.9700
C2—C3	1.390 (6)	C9—H92	0.9700
C3—C4	1.377 (6)	C10—H101	0.9700
C4—C5	1.377 (5)	C10—H102	0.9700
C4—C7	1.517 (4)	C11—H111	0.9700
C5—C6	1.385 (6)	C11—H112	0.9700
C7—C11	1.535 (4)	C12—H121	0.9700
C7—C8	1.528 (4)	C12—H122	0.9700
C8—C9	1.515 (5)		
			
C12—O1—H1	118 (6)	C4—C5—H5	119.00
C9—N1—C10	114.0 (3)	C6—C5—H5	119.00
C9—N1—H12	105 (5)	C1—C6—H6	121.00
C10—N1—H11	107 (3)	C5—C6—H6	121.00
H11—N1—H12	106 (6)	C4—C7—H7	107.00
C10—N1—H12	119 (5)	C8—C7—H7	107.00
C9—N1—H11	105 (3)	C11—C7—H7	107.00
F1—C1—C2	118.7 (5)	C7—C8—H8	108.00
F1—C1—C6	118.5 (5)	C9—C8—H8	108.00
C2—C1—C6	122.8 (4)	C12—C8—H8	108.00
C1—C2—C3	118.7 (5)	N1—C9—H91	109.00
C2—C3—C4	121.2 (4)	N1—C9—H92	109.00
C3—C4—C7	123.1 (3)	C8—C9—H91	109.00
C5—C4—C7	119.4 (3)	C8—C9—H92	109.00
C3—C4—C5	117.5 (3)	H91—C9—H92	108.00
C4—C5—C6	122.5 (5)	N1—C10—H101	109.00
C1—C6—C5	117.1 (4)	N1—C10—H102	109.00
C8—C7—C11	109.0 (2)	C11—C10—H101	109.00
C4—C7—C11	112.3 (3)	C11—C10—H102	110.00
C4—C7—C8	113.9 (2)	H101—C10—H102	108.00
C9—C8—C12	108.7 (3)	C7—C11—H111	110.00
C7—C8—C9	109.4 (2)	C7—C11—H112	110.00
C7—C8—C12	113.5 (3)	C10—C11—H111	110.00
N1—C9—C8	113.5 (3)	C10—C11—H112	110.00
N1—C10—C11	110.7 (3)	H111—C11—H112	108.00
C7—C11—C10	110.4 (3)	O1—C12—H121	110.00
O1—C12—C8	108.3 (3)	O1—C12—H122	110.00
C1—C2—H2	121.00	C8—C12—H121	110.00
C3—C2—H2	121.00	C8—C12—H122	110.00
C2—C3—H3	119.00	H121—C12—H122	108.00
C4—C3—H3	119.00		
			
C10—N1—C9—C8	51.5 (5)	C5—C4—C7—C11	−103.8 (4)
C9—N1—C10—C11	−52.1 (6)	C4—C5—C6—C1	0.8 (6)
F1—C1—C2—C3	−178.5 (4)	C4—C7—C8—C9	−176.0 (3)
C6—C1—C2—C3	2.4 (8)	C4—C7—C8—C12	−54.5 (4)
F1—C1—C6—C5	178.4 (4)	C11—C7—C8—C9	57.7 (4)
C2—C1—C6—C5	−2.5 (7)	C11—C7—C8—C12	179.3 (3)
C1—C2—C3—C4	−0.5 (7)	C4—C7—C11—C10	172.4 (3)
C2—C3—C4—C5	−1.1 (6)	C8—C7—C11—C10	−60.4 (4)
C2—C3—C4—C7	178.6 (4)	C7—C8—C9—N1	−53.7 (4)
C3—C4—C5—C6	1.0 (5)	C12—C8—C9—N1	−178.2 (3)
C7—C4—C5—C6	−178.8 (3)	C7—C8—C12—O1	−58.0 (4)
C3—C4—C7—C8	−48.1 (5)	C9—C8—C12—O1	64.0 (4)
C3—C4—C7—C11	76.5 (4)	N1—C10—C11—C7	56.7 (5)
C5—C4—C7—C8	131.7 (3)		

### Hydrogen-bond geometry (Å, °)

**Table:**

*D*—H···*A*	*D*—H	H···*A*	*D*···*A*	*D*—H···*A*
O1—H1···Cl1^i^	0.81 (7)	2.35 (7)	3.114 (4)	160 (6)
N1—H11···Cl1	0.83 (5)	2.56 (5)	3.234 (5)	140 (4)
N1—H12···Cl1^ii^	0.84 (5)	2.41 (5)	3.144 (5)	147 (6)

Symmetry codes: (i) *x*−1, *y*, *z*; (ii) *x*, *y*+1, *z*.
